# Supplementary material for: HIV Pretreatment Drug Resistance Trends in Mexico City, 2017–2020
Source: Pathogens. 2021 Dec 8;10(12):1587. doi: 10.3390/pathogens10121587 (PMC8708254; doi:10.3390/pathogens10121587)
Supplement: Supplementary file 1 [file pathogens-10-01587-s001.zip › pathogens-1466968-supplementary.pdf]

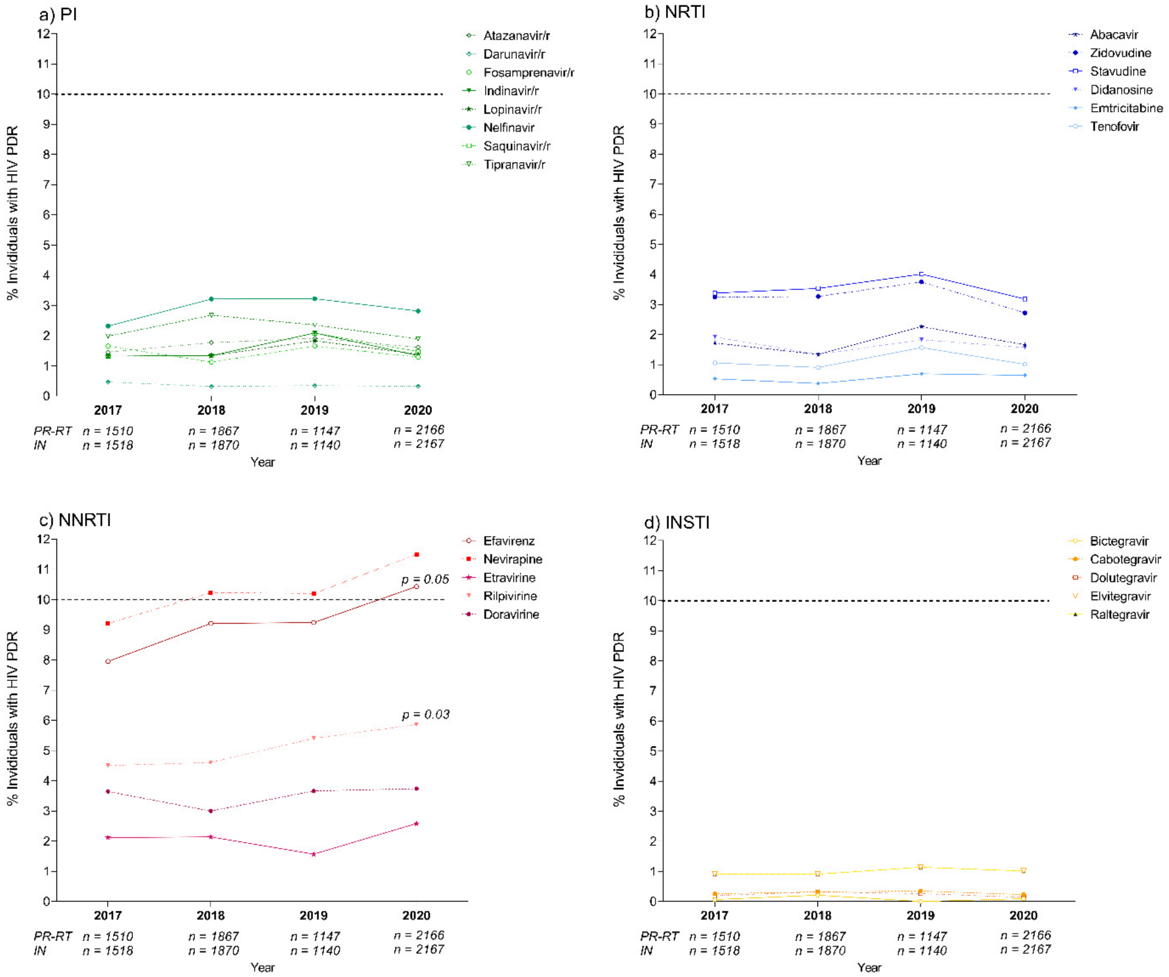

Figure S1. Pretreatment drug resistance trends in Mexico City by antiretroviral drug, 2017–2020. Antiretroviral drugs are grouped by drug class: (**a**) PI, (**b**) NRTI, (**c**) NNRTI, and (**d**) INSTI. HIV drug resistance was estimated from PR, RT and IN sequences using the Stanford HIVdb tool (v9.0). Trends were tested using linear regression. PDR, pretreatment drug resistance; PI, protease inhibitors; NRTI, nucleoside analogue reverse transcriptase inhibitors; NNRTI, non-nucleoside analogue reverse transcriptase inhibitors; INSTI, integrase strand transfer inhibitors.

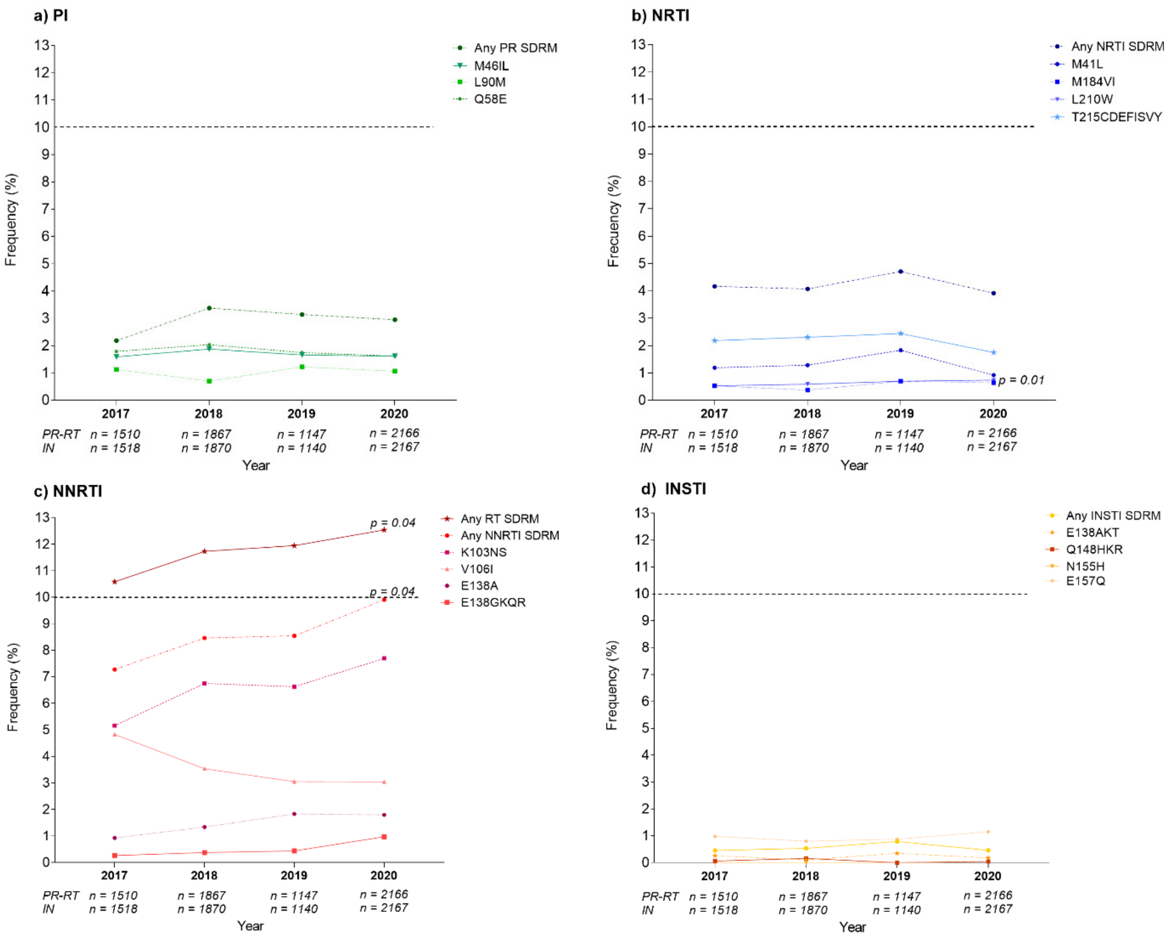

Figure S2. Pretreatment antiretroviral drug resistance mutation frequency trends in Mexico City, 2017–2020. Mutations are classified by drug class: (**a**) PI, (**b**) NRTI, (**c**). NNRTI, and (**d**) INSTI. Trends were tested using linear regression. Selected DRMs including both SDRM and non-SDRM are shown. PI, protease inhibitors; NRTI, nucleoside analogue reverse transcriptase inhibitors; NNRTI, non-nucleoside analogue reverse transcriptase inhibitors; INSTI, integrase strand transfer inhibitors; DRM, drug resistance mutation; SDRM, surveillance DRM.

Table S1. Demographic, clinical and behavioral characteristics of antiretroviral treatment restarters [a].

	ARV Naïve [b] (n = 1145)	Prior ARV Exposure [b] (n = 141)	p Value
Gender, *n* (%)			
Cisgender Women	64 (5.6)	18 (12.8)	
Cisgender Men	1060 (92.6)	115 (81.6)	<0.001 *
Transgender Women	21 (1.8)	7 (5.0)	
Transgender Men	0 (0.0)	1 (0.7)	
Age (years), *n*			
Median (IQR)	29 (25–35)	35 (29–41)	<0.001 *
Viral load (log RNA copies/mL), *n*			
Median (range)	4.7 (4.1–5.2)	4.4 (3.8–4.9)	0.0013 *
CD4+ T cell count category, *n* (%)			
<200 cells/mm^3	642 (56.1)	81 (57.5)	
200–500 cells/mm^3	449 (39.3)	53 (37.6)	0.905
>500 cells/mm^3	53 (4.6)	7 (5.0)	
Sexual risk category, *n* (%)			
Heterosexual cis women	59 (5.2)	18 (12.8)	
Heterosexual cis men	147 (12.8)	23 (16.3)	0.001 *
Cisgender MSM	782 (68.3)	85 (60.3)	
Transgender women	25 (2.2)	6 (4.3)	
Missing	132 (11.5)	9 (6.9)	
Marital status, *n* (%)			
Single	828 (72.3)	104 (73.8)	
Married	36 (3.1)	9 (6.4)	0.001 *
Domestic partnership	150 (13.1)	21 (14.9)	
Other	24 (2.1)	5 (3.6)	
Missing	107 (9.3)	2 (1.4)	
Education, *n* (%)			
Illiterate	5 (0.4)	2 (1.4)	
Elementary	44 (3.8)	15 (10.6)	
High School	499 (43.6)	70 (49.6)	<0.001 *
Technician	57 (5.0)	7 (5)	
Degree	388 (33.9)	42 (29.8)	
Postgraduate	40 (3.5)	3 (2.1)	
Missing	112 (9.8)	2 (1.4)	
Self-identified social class, *n* (%)			
Low	405 (35.4)	61 (43.3)	
Middle	623 (54.4)	77 (54.6)	0.004 *
High	2 (0.2)	0 (0.0)	
Missing	115 (10.0)	3 (2.1)	
Other sexually transmitted infections, *n* (%) [c]			
No	554 (48.4)	67 (47.5)	
Yes	361 (31.5)	58 (41.3)	0.024 *
Preferred not to answer	5 (4.4)	5 (3.5)	
Missing	180 (15.7)	11 (7.8)	
Role in anal sex, *n* (%)			
Receptive	277 (24.2)	34 (24.1)	
Insertive/Receptive	419 (36.6)	44 (31.2)	0.502
Insertive	195 (17.0)	25 (17.7)	
Missing	254 (22.2)	38 (27.0)	
Circumcision, *n* (%)			
No	704 (61.5)	84 (59.6)	0.766
Yes	211 (18.4)	25 (17.7)	
Missing	230 (20.1)	32 (22.7)	
Injectable drug use, *n* (%)			
No	992 (86.6)	124 (87.9)	0.001 *
Yes	36 (3.1)	12 (8.5)	
Missing	5117(10.2)	5 (3.6)	
Drug use for sex, *n* (%)			
No	540 (47.2)	73 (51.7)	0.135
Yes	439 (38.3)	56 (39.7)	
Missing	166 (14.5)	12 (8.5)	

Use of Apps for finding sexual partners, *n* (%)			
No	413 (36.1)	60 (64.6)	0.042 *
Yes	490 (42.8)	45 (31.9)	
Missing	242 (21.1)	36 (25.5)	

[a] Data collected for a subset of 1348 participants enrolled from June to December 2020. [b] Column percentages are shown. [c] During the previous six months. * $p < 0.05$. ARV, antiretroviral; MSM, men who have sex with men.
